# Treatment of ischemic stroke with modified mesenchymal stem cells

**DOI:** 10.7150/ijms.74161

**Published:** 2022-06-27

**Authors:** Hao Chen, Liangfu Zhou

**Affiliations:** Department of Neurovascular Surgery, First Hospital of Jilin University, 1xinmin Avenue Changchun130021, Jilin Province, China.

**Keywords:** Mesenchymal stem cells, Modification, Ischemic stroke, treatment

## Abstract

Ischemic stroke is one of the leading causes of death and disability. Ischemia triggers a cascade of events leading to cell death and cerebral infarction. Mesenchymal stem cell (MSC) therapy is a promising treatment modality to promote the development of nerve and blood vessels and improve nerve function. However, MSCs have a limited therapeutic effect in the harsh microenvironment of ischemic brain tissue. Modified MSC therapy shows better therapeutic effect under different pathological conditions, and is expected to be translated into clinical practice. In this article, we review the latest advances in the development of modified MSCs for the treatment of cerebral ischemia. In particular, we summarize the targets involved in migration, homing, antioxidant stress, anti-inflammatory, nerve and vascular regeneration, providing new ideas for clinical transformation.

## Introduction

Stroke is the second leading cause of death in the world after ischemic heart disease 1. Ischemic stroke (IS) accounts for 87% of all stroke patients, and its incidence rate is still rising 2, 3. IS is also a leading cause of disability. In China, IS accounts for approximately 70% of all cases of stroke 4. An estimated three quarters of patients with ischemic stroke drop out of the labor force, and two fifths of these patients develop severe disability 5. Therefore, prevention and treatment of ischemic stroke is a key research imperative.

Currently, there is a paucity of effective treatments for IS. Tissue plasminogen activator (T-PA) is administered intravenously to unblock the blocked blood vessels. However, the time window for T-PA treatment is short (≤4.5 hours) and there is a risk of secondary intracerebral hemorrhage 6. Mechanical thrombectomy (MT) can extend the treatment window to 24 hours, but this particular procedure can only be performed in a limited number of qualified hospitals and requires strict screening for indications and contraindications; therefore, only a few patients are able to receive MT treatment 7. Therefore, it is imperative to develop a new treatment method for IS.

Mesenchymal stem cells (MSCs) are multipotent cells that can specialize into several cell types from different lineages. Intravenously administered MSCs can migrate to the site of tissue damage and promote angiogenesis, growth, and differentiation of local progenitor cells 8-12. Some studies have shown no significant risk of host immune response to allogeneic transplantation of MSCs 13-15. In addition, MSCs are easy to isolate and culture from different tissues such as cord blood, bone marrow, and adipose tissue. These attributes make MSCs the main source of cell therapy for many diseases.

MSC therapy has been shown to promote post-stroke functional recovery and neurological outcomes 16, 17. In addition, clinical trials of MSC therapy for the treatment of IS have demonstrated its safety and feasibility 18-22. However, there are several barriers that limit its use and therapeutic effectiveness. For example, in the harsh microenvironment of stroke (inflammation storm, oxidative stress), isolated MSCs gradually lose their homing ability to the lesion 23. In clinical trials, although MSC therapy was shown to be safe and confer some therapeutic effects, the effects were not significant 24-26. Attempts have been made to develop novel MSC-based methods for the treatment of IS, such as genetically-modified MSCs, and use of preconditioning, electrical stimulation, and ultrasonic stimulation. A large number of studies published in recent years have demonstrated the improved ability of MSCs in the treatment of IS by using genetic modification or preconditioning of MCS in combination with physical therapy 23, 27-30. This paper provides an overview of the recent advances in this field.

## Application of modified MSCs in the treatment of ischemic stroke

**Figure 1:**
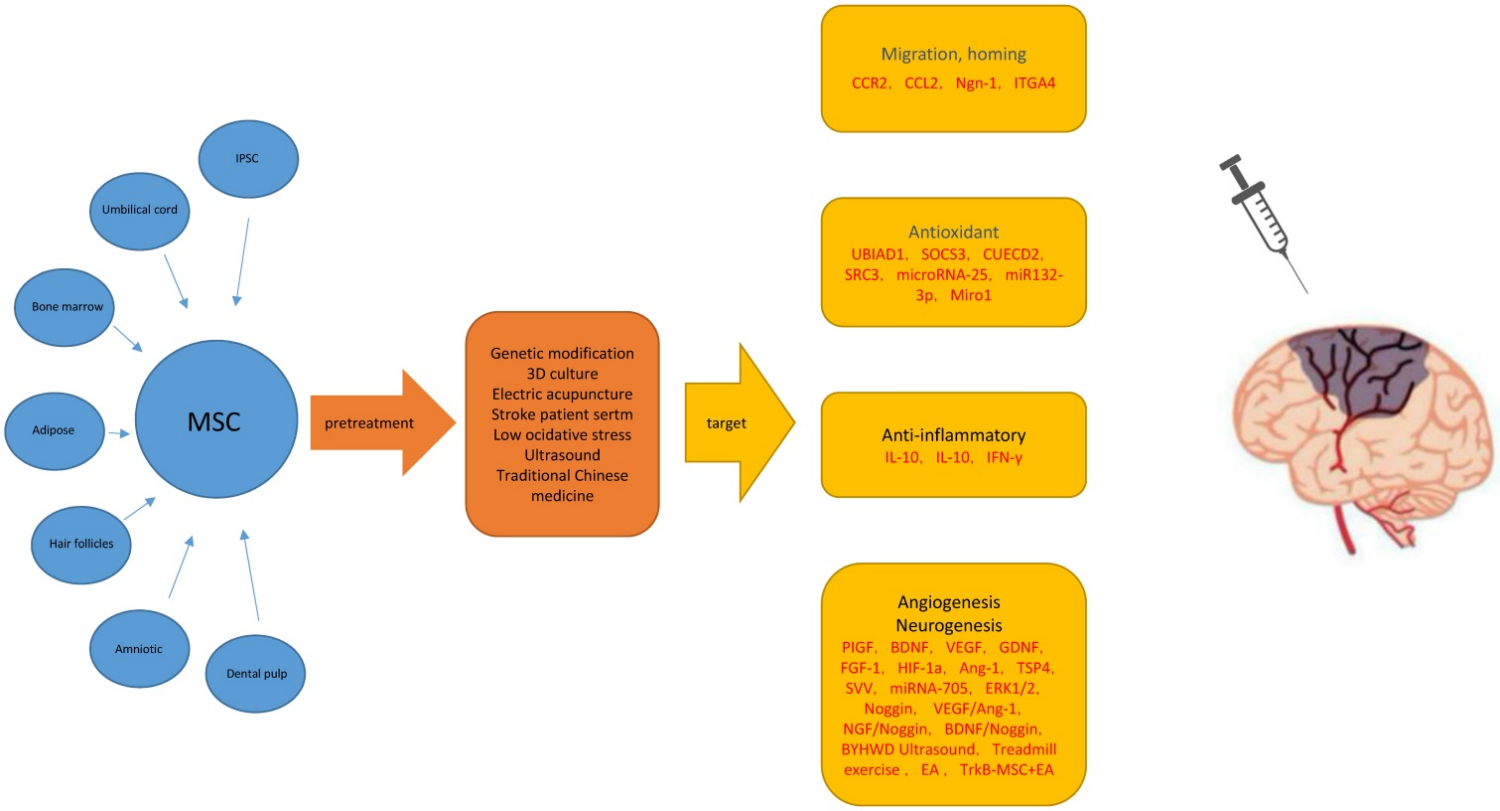
Possible main mechanisms for improving the therapeutic effect of mesenchymal stem cells in ischemic stroke models. **Abbreviations:** MSC: Mesenchymal Stem Cells; ROS: Reactive Oxygen Species; t-PA: tissue plasminogen activator; BMSCs: bone marrow mesenchymal stem cells; UMSCs: umbilical cord stromal cells; ADSC: Adipogenic stromal cells; MCAO: Middle cerebral artery occlusion; BBB: blood brain barrier.

There are several therapeutic challenges in the use of MSC therapy for treatment of IS. The key issues include whether MSCs and their exosomes (microvesicles) can migrate to the target organ and play a role, their ability to survive for a long time in the ischemic and hypoxic brain tissue, and their ability to successfully transform into functional nerve cells in the damaged area (Figure 1).

### Migration and Homing

It is generally believed that the homing ability of MSCs at the site of target lesions and their implantation determines the therapeutic effect of MSC therapy 31, 32. However, the decreased expression of some chemokine receptors (such as CCR2 and CXCR4) during the continuous passage of MSC was found to affect the homing ability of MSCs to target lesions 33-39. In order to improve the homing ability of MSCs, it was found that the expression of some chemokines (CCR1, CCR2, CXCR4) was increased after gene modification or pretreatment of MSCs 37, 40. At the same time, the interaction of CCL2/CCR2 and SDF-1/CXCR4 was shown to significantly improve the homing and migration of modified MSCs during acute ischemic attack 41-43. This was also shown to significantly improve the neurological function 23, 44-50.

Secondly, in the experimental middle cerebral artery occlusion (MCAO) model, only a small part of intravenously injected MSCs entered the ischemic brain tissue, and most of them were trapped in the lung and spleen 33, 51, which also affected the homing of MSCs. Compared with intravenous administration, MSCs were found to more readily migrate to the damaged brain tissue after arterial injection; in addition, genetically-modified MSCs were found to survive longer in the ischemic brain tissue 40 and reduce vascular embolization 43. Exosomes and microvesicles of MSCs are small in size and more readily pass through the lung tissue; in addition, these contain many molecules that may have therapeutic effects on stroke 52, 53. However, extractable exosomes and microvesicles require large amounts of MSCs for therapeutic purposes. MSC culture methods can be modified, such as by using microcarriers and hollow fiber bioreactors to culture MSCs in a 3D environment, so that they can be massively amplified 54. This can serve the purpose of treatment.

### Antioxidant

During brain ischemia and hypoxia, the adverse microenvironment induced by excessive oxidative stress leads to the death of a large number of transplanted MSCs, which further hinders the therapeutic effect of MSC therapy 33, 55, 56. Oxidative stress results from the excessive production of reactive oxygen species (ROS), which triggers many cellular and molecular events, leading to the oxidation of proteins and lipids and ultimately to neuronal death 57-59. Mitochondria are the main organelles responsible for ROS production 60. Therefore, oxidant/antioxidant imbalance and mitochondrial dysfunction are the basic triggers of neuronal injury in IS. Studies have shown that some target genes (*UBIAD1, SOCS-3, CUEDC2, SRC3*) or specific miRNAs (microrNA-25, Mir-132-3p) can target specific antioxidant enzymes 61 or activate the PI3K/Akt/eNOS pathway 62. This can increase the ratio of antioxidant enzyme to oxidase and inhibit oxidative stress reaction 28, 29, 63, 64, thus enabling MSCs or their exosomes to obtain a stronger antioxidant effect. In addition, when neurons and astrocytes are exposed to excessive ROS, mitochondria more efficiently transfer from mesenchymal stem cells to the damaged cells 65, 66. Interestingly, mitochondrial movement from MSCs to damaged brain regions during oxidative stress was enhanced through genetic modification of MSCs 67. At the same time, different types of MSCs have different adaptability in the harsh environment of oxidative stress. For example, umbilical cord derived mesenchymal stem cells showed more adaptability 68.

### Anti-inflammatory

In the acute stage of cerebral ischemia, the progression of cerebral infarction and the formation of cerebral edema are closely related to the strong inflammatory response. It is characterized by rapid microglial activation, production of pro-inflammatory mediators, and infiltration of inflammatory cells into injured brain tissue 69, 70. The anti-inflammatory effects of MSCs are characterized by down-regulation of secretion of anti-inflammatory molecules by pro-inflammatory cytokines 71, prevention of leukocyte infiltration 72, and promotion of polarization of the M2 phenotype of microglia 73. Inhibition of inflammation can stabilize blood brain barrier (BBB) function and inhibit neuronal apoptosis.

As mentioned above, MSCs play an anti-inflammatory role by secreting IL-6 and reducing the pro-inflammatory factor TNF-α. This signaling pathway may be related to the inhibition of NF-κB by MSCs 74. Meanwhile, the immunomodulatory cytokine IL-23/IL-17 of MSCs play a role in ischemic stroke 75.

MSCs can induce pro-inflammatory M1 microglia to differentiate into anti-inflammatory M2 microglia after IS. It has been reported that MSCs cause low expression of microglia activation markers (ED1 and Iba) and astrocyte proliferation markers (GFAP) 76. These results suggest that the immunomodulatory effect of MSCs may be related to the inhibition of microglia and astrocytes residing in the brain, which may be related to the non-phosphorylation of STAT3 in the atypical JAK-STAT signaling pathway 77.

MSCs can reduce the release of neutrophil matrix metalloproteinase-9 (MMP-9), maintain the integrity of the blood-brain barrier, and reduce the infiltration of inflammatory cells in brain parenchyma 78. In addition, MSCs reduce Monocyte chemotactic protein-1 (McP-1) production by secreting the anti-inflammatory cytokine TGF-β, thereby blocking the migration of CD68 + immune cells to ischemic regions 79.

Overexpression of anti-inflammatory factors enhanced the anti-inflammatory effect of MSCs, leading to enhanced neuroprotective function. In addition, IL-10 overexpressed MSCs can delay the time window for MSC therapy without affecting serum IL-10 levels, which may reduce the risk of systemic IL-10-induced adverse reactions such as anemia, thrombocytopenia, and immunosuppression 27, 80-82. At the same time, MSCs activated by interferon gamma showed a better effect in the treatment of acute IS, resulting in a significant reduction of CD68 + monocytes and microglia 83 (Table 1).

### Neurogenesis and Angiogenesis

It is generally believed that the paracrine effect of MSCs plays a role in endogenous neural differentiation and proliferation 83. Although endogenous neural stem cells (NSCs) do exhibit an acute response to IS, such as increased cell proliferation and cell migration, only 10%-20% of these cells survive long-term, and only a few of these surviving cells can mature into functional cells. However, most of them develop into thorny new striatal projection neurons or calretinin-positive interneurons 84. This hinders the differentiation of MSCs into functional nerve cells after transplantation. Neural factors are known to play an important role in neurogenesis and vasculogenesis. MSCs genetically modified with neural factors can significantly increase the content of FGF, BDNF, VEGF, NGF, PIGF, and GDNF in the ischemic brain tissue, and promote the differentiation and proliferation of NSCs in the SVZ region during treatment. In addition to the generation of blood vessels, it promotes the generation of mature nerve cells and improves nerve function 85-92. Some targeted miRNAs also play an important role in functional neurogenesis 93, 94. MSCs overexpressing mirNA-133B were shown to regulate CTGF expression in astrocytes and RhoA expression in the IBZ region, promoting neurite remodeling and improving functional recovery in MCAO rats 94. In addition to the chemical methods, use of physical methods may also play an important role. In a study, use of ultrasound probe to stimulate the ischemic brain tissue was shown to increase the expression of P-ERK and P-CREb and significantly promote neurogenesis 95. In addition, the combination of chemical and physical methods significantly improved the therapeutic effect of modified MSCs on neurogenesis and angiogenesis. Studies have shown that the combination of overexpression of TRKB in MSCs and electroacupuncture stimulation may result in successful transdifferentiation of transplanted MSCS into functional nerve cells 96.

In conclusion, modification of MSCs by sensitive targets has shown a more significant effect in the treatment of IS stroke than MSCs alone.

## Clinical research status of modified mesenchymal stem cells

Clinical trials have demonstrated the safety of MSC therapy in IS. MSCs derived from allofat were found to be safe for treatment in the acute phase of ischemia 97, and a Phase IIa clinical trial (NCT01678534) has been completed. Other clinical trials of MSC allografts are also being recruited (NCT04811651) (NCT05008588) (NCT04280003) (NCT03384433) (NCT04434768) (NCT04590118) (NCT02580019) (NCT04093336). In addition, the safety of hypoxic-treated allograft BM-MSCs has been demonstrated in the treatment of chronic stroke. In addition, there was significant improvement in behavioral endpoints 98 (NCT01297413). This evidence makes us look forward to the transformation of MSCs.

Some experimental studies suggest the feasibility of use of modified MSCs for the treatment of IS. In particular, genetically modified MSCs have shown promising therapeutic effects, but not much work has been done in clinical transformation. The key challenges to clinical transformation include the cytotoxicity of vectors such as lentiviruses, adenoviruses or retroviruses, and carcinogenicity and immunogenicity of viral DNA integration into the host genome. However, clinical studies in the context of other diseases have demonstrated the safety of the treatment process and good results have been achieved. For example, in a clinical study of neuroblastoma 99, autologous MSCs injected with ICoVIR-5 (a novel oncolytic adenovirus) for the treatment of metastatic neuroblastoma was found to be safe and effective. In addition, the use of transgenic autologous MSCs has been shown to improve the targeting of tumor cells in the treatment of gastrointestinal tumors 100. In addition to neoplastic diseases, there have been some clinical trials of inducing MSCs to secrete target proteins in degenerative diseases of the nervous system. Brain-Storm Cell Therapeutics concluded a phase I/IIa clinical trial in patients with amyotrophic lateral sclerosis (ALS) using autologous MSCs induced to express neurotrophic factor (NurOwn) with mild and transient adverse effects reported. Strikingly, treated ALS patients demonstrated slowed disease progression following the conclusion of the Phase IIa trial with improvements in breathing and reduced motor decline compared to pre-treatment level 101 (NCT01051882) (NCT01777646). In addition, clinical studies evaluating MSC/BDNF in a dose-dependent manner to demonstrate the safety of transgenic MSCs for striate injection transplantation in patients with HD are being observed 102 (NCT01937923). In a 2-year 1/2A study, Gary K Steinberg and his team implanted modified bone marrow MSCs (SB623) into chronic ischemic brain tissue using transient transfection of human Notch-1 intracellular domain. They concluded that SB623 cell implantation in patients with stable chronic stroke is safe and accompanied by improved clinical outcomes 103 (NCT01287936).

In conclusion, allogeneic MSC therapy for IS appears safe and feasible. The safety and effectiveness of genetically-modified MSCs has been demonstrated in clinical trials. The available evidence suggests a promising outlook of the use of various gene targets or preconditioning of modified allogeneic MSCs for the treatment of IS at all stages.

## Outlook

Advances in the field of biotechnology have helped improve the treatment of a wide range of diseases. In recent years, the development of crisPR-Cas9 and other gene technologies has made rapid progress in the treatment of metabolic diseases and cancers. Modification of MSCs appears a particularly promising approach as a therapeutic modality. In conclusion, modification of MSCs for ischemic stroke to enhance their targeting ability is a feasible and highly applicable research direction for future clinical transformation.

## Conclusion

Ischemic stroke is a disease characterized by high morbidity, disability, and mortality. Complex pathological changes occurring in the damaged brain tissue including inflammatory storm, oxidative stress, and nerve cell apoptosis lead to severe neurological dysfunction. MSC therapy is a promising treatment for IS. However, the harsh ischemic and hypoxic microenvironment limits the effectiveness of this treatment modality. Modification of MSCs to improve their therapeutic ability represents a feasible and applicable research direction in clinical transformation.

## Figures and Tables

**Table 1:** Target genes that promote migration, homing, antioxidant, anti-inflammatory ability of modified mesenchymal stem cells, MSC source, infusion mode, infusion time, and cell number

		Transplantation	Number of transplanted cells	Transplantation of time	Source of MSC	target
Homing and migration	23	IV	2X10 6	24 after MCAO	hBM-MSC	CCR2
	50	IV	1x10 6	1 and 4 days after MCAO	hUC-MSC	CCL2
	40	IA	1x10 6	3 days after MCAO	hBM-MSC	Ngn-1
	43	IA	5X10 5	24 h after MCAO	rBM-MSC	ITGA4
Antioxidant stress	63	IV	5X10 6	24 h after MCAO	OM-MSC	UBIAD1
	28	IV	2X10 6	3 h after MCAO	rBM-MSC	SOCS3
	29	ICV	2X10 6	24 h after MCAO	rBM-MSC	CUECD2
	64				rBM-MSCs	SRC3
	61	Intrathecal Injection	20ug	1day before Ischemia	BM-MSCs	microRNA-25
	62	IV	1 × 10^10^ particles/100 μL in PBS	90min after MCAO	BM-MSCs	miR-132-3p
	67	IV	3 × 10^6^ cells/kg	-	hMMSC	Miro1
Anti-inflammatory	82	IV	2X10 6	3 h after MCAO	rBM-MSC	IL-10
	27	IV	1x10 6	0 or3 h after MCAO	hBM-MSC	IL-10
	83	IV	5×106 cells/kg	3 h after MCAO	BM-MSC	IFN-γ
Neurogenesis, Angiogenesis	96	ICV	1x10^6^/2ul	5d after MCAO	BM-MSC	TrkB-MSC+EA
	90	IV	1x10 7	3 h after MCAO	hBM-MSC	PIGF
	87	ICV	5X10 5	24 h after MCAO	hBM-MSC	BDNF
	91	ICV	1x10 6	24 h after MCAO	rBM-MSC	VEGF
	88	IV	1x10 7	3 h after MCAO	hBM-MSC	GDNF
	86	IV	2X10 6	30 min after MCAO	AD-MSC	FGF-1
	30	IV	1x10 6	6 h after MCAO	hBM-MSC	Ang-1
	93	IA	2x10 5	24 h after MCAO	mBM-MSC	miRNA-705
	92	IV	5X10 6	6 h after MCAO	rBM-MSC	Noggin
